# Glutamine Attenuates Acute Lung Injury Caused by Acid Aspiration

**DOI:** 10.3390/nu6083101

**Published:** 2014-08-05

**Authors:** Chih-Cheng Lai, Wei-Lun Liu, Chin-Ming Chen

**Affiliations:** 1 Department of Intensive Care Medicine, Chi Mei Medical Center, Liouying Dist., Tainan 73657 Taiwan; E-Mails: dtmed141@gmail.com (C.-C.L.); medrpeterliu@gmail.com (W.-L.L.); 2 Department of Recreation and Health-Care Management, Chia Nan University of Pharmacy and Science, Tainan 71710, Taiwan; 3 Chang Jung Christian University, Tainan 71101, Taiwan; 4 Departments of Intensive Care Medicine, Chi Mei Medical Center, Tainan, 71073, Taiwan

**Keywords:** acid aspiration, ARDS, glutamine, ventilator-induced lung injury

## Abstract

Inadequate ventilator settings may cause overwhelming inflammatory responses associated with ventilator-induced lung injury (VILI) in patients with acute respiratory distress syndrome (ARDS). Here, we examined potential benefits of glutamine (GLN) on a two-hit model for VILI after acid aspiration-induced lung injury in rats. Rats were intratracheally challenged with hydrochloric acid as a first hit to induce lung inflammation, then randomly received intravenous GLN or lactated Ringer’s solution (vehicle control) thirty min before different ventilator strategies. Rats were then randomized to receive mechanical ventilation as a second hit with a high tidal volume (TV) of 15 mL/kg and zero positive end-expiratory pressure (PEEP) or a low TV of 6 mL/kg with PEEP of 5 cm H_2_O. We evaluated lung oxygenation, inflammation, mechanics, and histology. After ventilator use for 4 h, high TV resulted in greater lung injury physiologic and biologic indices. Compared with vehicle treated rats, GLN administration attenuated lung injury, with improved oxygenation and static compliance, and decreased respiratory elastance, lung edema, extended lung destruction (lung injury scores and lung histology), neutrophil recruitment in the lung, and cytokine production. Thus, GLN administration improved the physiologic and biologic profiles of this experimental model of VILI based on the two-hit theory.

## 1. Introduction

Acute respiratory distress syndrome (ARDS) is a catastrophic syndrome among critically ill patients. One of its major causes is acid aspiration as an initial pneumonitis that may be complicated by subsequent bacterial pneumonia after inhaling low pH gastric fluid [1,2,3,4]. Gastric fluid aspiration frequently occurs in trauma or critical patients with head trauma, alcohol or cerebrovascular accidents, and is also a complication of general anesthesia that occurs in 1 in 2000–3000 cases when anesthetics are used [3,4].

ARDS results in pulmonary inflammation and hypoxemia, mechanical ventilation (MV) may be a necessary as a life-saving treatment [2]. However, inadequate MV settings may cause mechanical stress that can result in ventilator-induced lung injury (VILI). Generation of an enhanced inflammatory response can also worsen lung injury [2,5]. The activation of nuclear factor (NF)-κB with the subsequent transcription of inflammatory mediator genes has been documented in the context of VILI [6]. The spectrum of injuries involved includes disruption of endothelial and epithelial cells, neutrophil infiltration, enhanced production of inflammatory cytokines, including interleukin (IL)-1β, IL-6, and tumor necrosis factor (TNF)-α, and subsequent permeability pulmonary edema, hyaline membranes, and decreased lung compliance [7].

A “two-hit injury” can be caused by VILI preceded by hemorrhagic shock and followed by reperfusion or acid aspiration, which makes the lung more susceptible to MV injury [8,9]. Although using a low tidal volume improves the clinical outcomes of certain populations of patients with ARDS, the mortality of ARDS patients remains high [10]. In addition, many conscious patients with ARDS receive MV that is less “controlled” during their ICU stay than that while in the operating room, which makes a protective lung strategy challenging [11,12]. Thus, therapeutic approaches in addition to using protective MV strategies are needed for managing ARDS.

Glutamine (GLN) is an important energy source for cell proliferation and a conditional essential amino acid that is the most abundant in the body [13]. Numerous studies have shown that GLN has immune modulation properties, as it attenuates the release of TNF-α, IL-1β, IL-6, and IL-8 in response to oxidative stress, and prevents lung injury along with improved outcomes in ARDS [14,15,16,17,18]. GLN exerts protective effects include its antioxidant effects by preserving reduced Glutathione (GSH) levels and inducing heat shock protein 70 via O-linked glycosylation-dependent activation of hexosamine biosynthesis [19,20]. This heat shock protein can enhance cell survival and attenuate the systemic inflammatory response associated with lung injury [21]. In particular, the enhanced levels of tissue GSH and heat shock proteins may be partially responsible for the attenuated cytokine responses [22] and the inhibition of NF-κB [23,24].

In this study, we established a model in rats with acid aspiration primed lung inflammation followed by VILI as a two-hit injury. We then assessed the potential benefits of GLN administration on this two-hit injury model by examining lung mechanics, blood gases, systemic inflammatory responses, and lung injuries.

## 2. Experimental Section

### 2.1. Rat Preparation

The Institutional Animal Care and Use Committee of Chi-Mei Hospital approved our experimental protocols (CMFHR10249, date of approval: 1 July 2013). Male Sprague-Dawley rats (300–400 g) were anesthetized by intraperitoneal injection of urethane (2 mg/kg; Sigma, St. Louis, MO, USA) while in the supine position. We used a 14-gauge cannula (Angiocath IV Catheter, 2.1 × 48 mm; Becton Dickinson Infusion Therapy Systems, Inc., Sandy, UT, USA) and inserted this into the trachea to create a tracheostomy. Hydrochloric acid (HCl, pH 2.0) at 0.4 mL/kg was instilled intratracheally using an intratracheal aerosolizer (Penn Century, Inc., Philadelphia, PA, USA). Immediately after acid administration, a recruitment maneuver was performed by increasing positive end-expiratory pressure (PEEP) levels to 25 cm H_2_O for 5 breaths using a Servo 300 ventilator (Siemens, Solna, Sweden). Subsequently, a tidal volume (TV) of 6 mL/kg, PEEP of 5 cm H_2_O, and a respiratory rate of 50 breaths/min with a fraction of inspired oxygen (FiO2) of 40% was administered. After ventilation for 5 min to stabilize a rat using these ventilator settings, an arterial blood gas sample was taken. PaO2 of about 150 mmHg was considered standard for a model of ARDS. The right carotid artery was cannulated (Angiocath IV Catheter; 24-gauge) to monitor mean arterial pressure (MAP) and to collect samples for blood gas analysis. An intravenous cannula was inserted into and maintained in the tail vein to maintain anesthesia by continuous infusion of ketamine (15 mg/kg/h), xylazine (3 mg/kg/h), and pancuronium (0.35 mg/kg/h) and infusion of lactated Ringer’s solution. We kept the MAP at >70 mmHg during the entire study period.

### 2.2. Experimental Protocol

Rats were observed for 30 min for stabilization after cannulation, and were then randomized into four groups (10 rats/group). Group 1: rats ventilated with high TV (HV); Group 2: rats ventilated with high TV and administered GLN (HVG); Group 3: rats ventilated with low TV (LV); and Group 4: rats ventilated with low TV and administered GLN (LVG). HV groups were ventilated with a TV of 15 mL/kg and zero PEEP at a respiratory rate of 16–18 breaths/min. LV groups were ventilated with a TV of 6 mL/kg and PEEP of 5 cm H_2_O at a rate of 45–55 breaths/min. FiO2 remained at 40%. We kept the ventilator period for 4 h after randomization.

### 2.3. GLN Administration

Either GLN with 20% Dipeptiven (Fresenius Kabi, Bad Homburg v.d.H., Germany) that contained 20 gm of *N*(2)-l-alanyl-l-glutamine or lactated Ringer’s solution used as vehicle control was administered as an i.v. bolus at 0.75 g/kg (3.75 mL/kg) at 30 min prior to randomizing for MV strategies (soon after inducing acid aspiration). The main purpose for choosing this time for GLN administration was to attenuate MV-induced lung injury because we knew the time when MV was applied.

### 2.4. Measurements

Rats were sacrificed by sodium pentobarbital overdose. Their lungs were excised via a midline sternotomy and static pressure-volume curves were generated by manually injecting 0.5 to 1 cm^3^ aliquots of air in a stepwise manner starting at atmospheric pressure and continuing until achieving an airway pressure of 30 cm H_2_O. We subsequently used a similar step-wise approach for deflation. At each step of air inflation or deflation, volumes were maintained for 6 s.

The left lung was lavaged (bronchoalveolar lavage, BAL) for cell differentiation determinations. The right upper lungs were used to measure wet-to-dry (W/D) lung weight ratios. Cytokine concentrations (IL-1β, IL-6, IL-10, CXCL1, and TNF-α) in plasma samples (*n* = 10/group) and BAL fluid samples (*n* = 10/group) were determined in a blinded fashion by technicians using DuoSet ELISA Development kits (R & D Systems, Minneapolis, MN, USA).

### 2.5. Lung Histology

The other parts of right lungs were used for histology and lung injury assessments, which were evaluated by a pathologist who was blinded to the experimental groups, based on methods described previously, including neutrophil counts (A) in the alveolar and (B) interstitial spaces (score 0 if no neutrophils; score 1 if 1–5 neutrophils; score 2 if >5 neutrophils); (C) hyaline membranes (score 0 if none; score 1 if 1 membrane; score 2 if >1 membranes); (D) proteinaceous debris that filled airspaces (score 0 if none; score 1 if 1 debris; score 2 if >1 debris); and (E) alveolar septal thickening (score 0 if <2 times; score 1 if 2–4 times; score 2 if >4 times) [25]. For each lung histology slide, five regions were examined. The score is calculated as [(20 × A) + (14 × B) + (7 × C) + (7 × D) + (2 × E)]/(number of fields × 100). The resulting injury score was a value between 0 and 1.

### 2.6. Statistical Analysis

Results are given as means ± standard errors of the mean. Group results were compared using one-way analysis of variance (ANOVA). *Post hoc* pair-wise comparisons were made using Bonferroni’s multiple comparisons test. Statistical analysis was done using SPSS 13.0 software (SPSS Inc. Chicago, IL, USA).The significance level was set at α = 0.05.

## 3. Results

**Figure 1:**
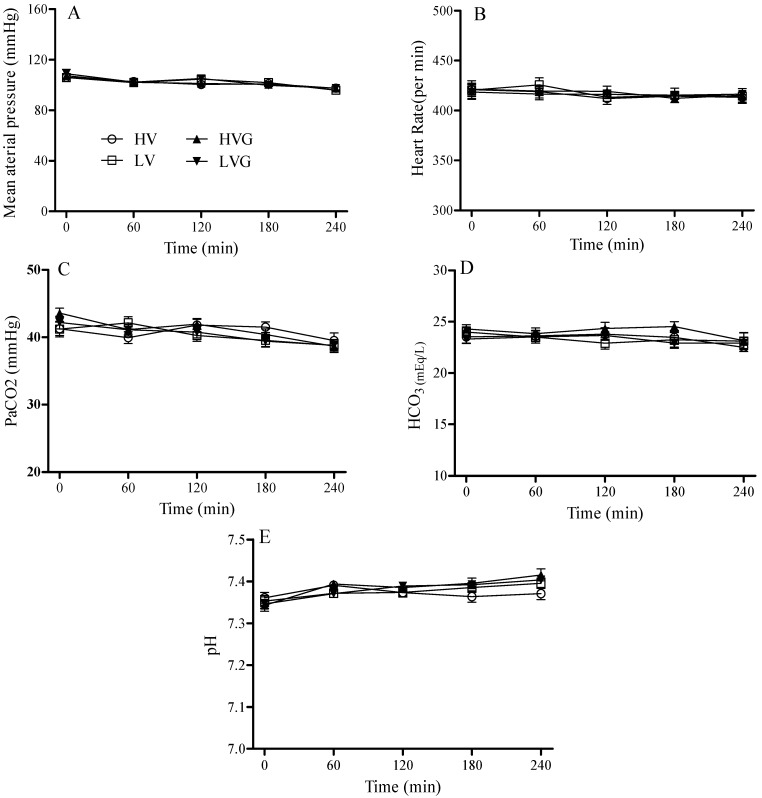
Hemodynamic status and blood gas analysis (PaCO_2_, HCO_3_, pH) during 4 h of mechanical ventilation after randomization. (**A**) Mean arterial pressure; (**B**) heart rate; (**C**) PaCO_2_; (**D**) HCO_3_; and (**E**) pH. Rat groups: HV, acid aspiration + high TV; LV, acid aspiration + low TV; HVG, acid aspiration + high TV + glutamine; LVG, acid aspiration + low TV + glutamine.

After ventilator use for 4 h, each group of rats had a similar hemodynamic status (MAP and heart rates) at baseline and during mechanical ventilation (Figure 1). The volumes of fluid that had been infused were identical (≈2.5 mL of lactated Ringer’s solution) for all rats during the mechanical ventilation period.

### 3.1. Blood Gas Analysis and Respiratory Mechanics

**Figure 2:**
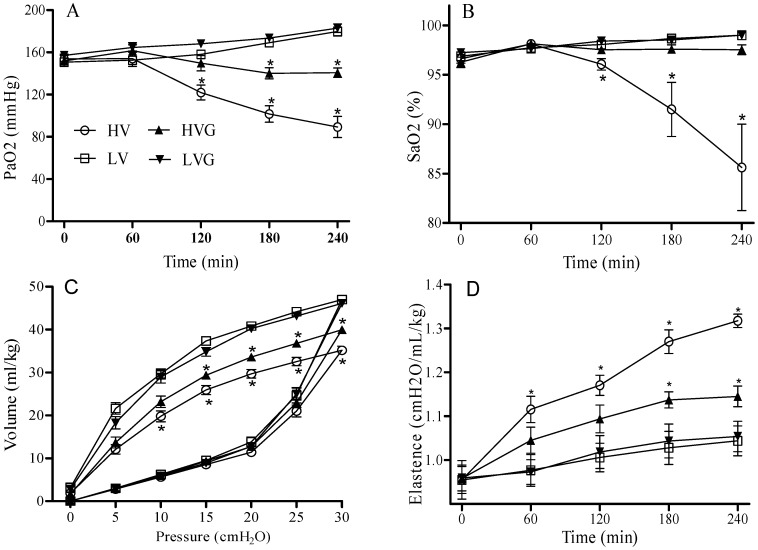
Arterial blood gas (**A**) PaO_2_ and (**B**) SaO_2_ during 4 h of mechanical ventilation after randomization. Static compliance curves (**C**) and lung elastance (**D**) at the end of the 4 h of mechanical ventilation. The bottom lines indicate inflation and the top line indicates deflation on (**C**). * *p* < 0.05 *vs.* other groups. Rat groups: HV, acid aspiration + high TV; HVG, acid aspiration + high TV + glutamine; LV, acid aspiration + low TV; LVG, acid aspiration + low TV + glutamine.

At baseline and during randomization for the two mechanical ventilator strategies, each group of rats also had similar arterial pH, PaCO_2_, and HCO_3_ values (Figure 1). The mean PaO_2_ and SaO_2_ values at baseline were similar for all rats (around 150 mmHg and 96%, respectively). However, these values were significantly lower in the HV group at 120 min after randomization, and the HV group also had lower levels at the end of the study period as compared with the other groups (Figure 2A,B). Thus, GLN administration had reversed this injury.

The HV group had the worst compliance among all groups at the end of lung expansion with 30 cm H_2_O based on static pressure-volume curves (Figure 2C). Similarly, lung elastance values at baseline were similar in all groups, although these increased significantly in the HV group compared to other groups at 60 min after randomization (Figure 2D). Again, GLN administration had attenuated these insults.

### 3.2. Cell Counts in Lung Lavage Fluids, Lung Wet-to-Dry Ratios, and Lung Injury Scores

**Figure 3:**
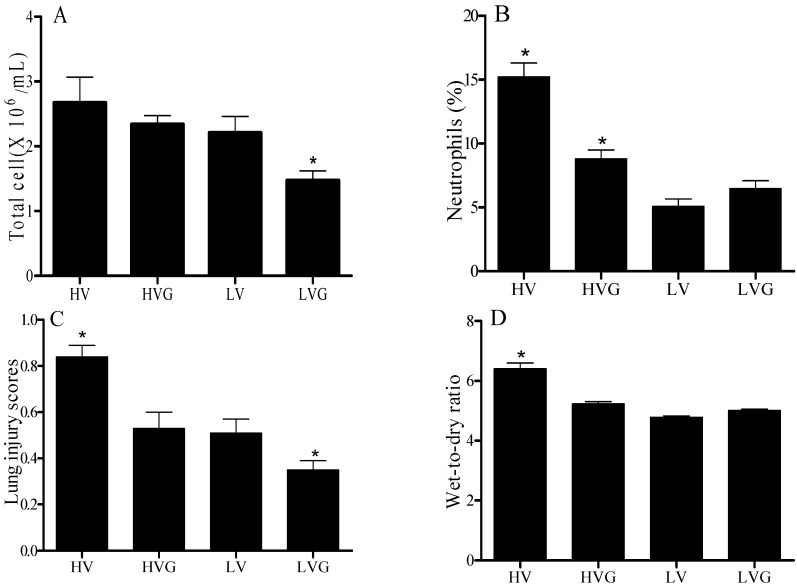
Cell counts in lung lavage (BAL) fluids, lung wet-to-dry ratios, and lung injury scores. (**A**) Total cells in BAL; (**B**) neutrophils (%) in BAL; (**C**) lung wet-dry ratios; and (**D**) lung injury scores. * *p* < 0.05 *vs.* other groups. Rat groups: HV, acid aspiration + high TV; HVG, acid aspiration + high TV + glutamine; LV, acid aspiration + low TV; LVG, acid aspiration + low TV + glutamine.

BAL fluid total cell counts (expressed as numbers × 10^6^/mL) were the highest in the HV group (2.67 ± 0.40) compared to the other groups (2.21 ± 0.26 in LV, 2.34 ± 0.14 in HVG, and 1.47 ± 0.15 in LVG; Figure 3A). Similarly, the HV group had a higher percentage of neutrophils in BAL fluid compared to the other groups (15.2% ± 1.1% *vs.* 5.1% ± 0.6% in the LV group, 8.8% ± 0.7 in the HVG group, and 6.5% ± 0.6% in the LVG groups; all *p* < 0.05; Figure 3B). The lung injury scores and the lung W/D ratios were also highest in the HV group compared to the other groups (Figure 3C,D). Thus, GLN administration had attenuated inflammation and lung injury after MV with a high TV (Figure 3).

### 3.3. Cytokine Profiles

**Figure 4:**
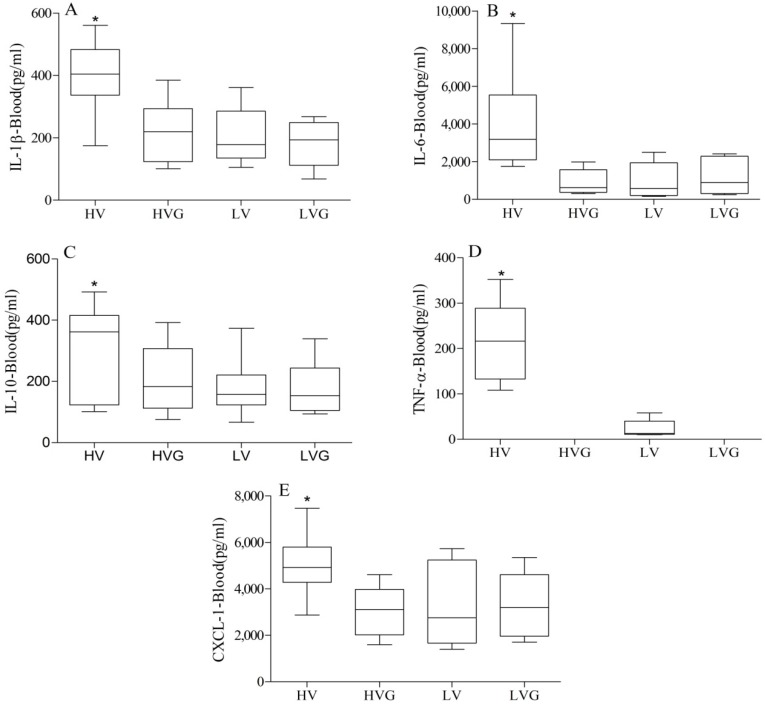
Plasma cytokine levels. * *p* < 0.05 *vs.* other groups (*n* = 10/group). Rat groups: HV, acid aspiration + high TV; HVG, acid aspiration + high TV + glutamine; LV, acid aspiration + low TV; LVG, acid aspiration + low TV + glutamine.

**Figure 5:**
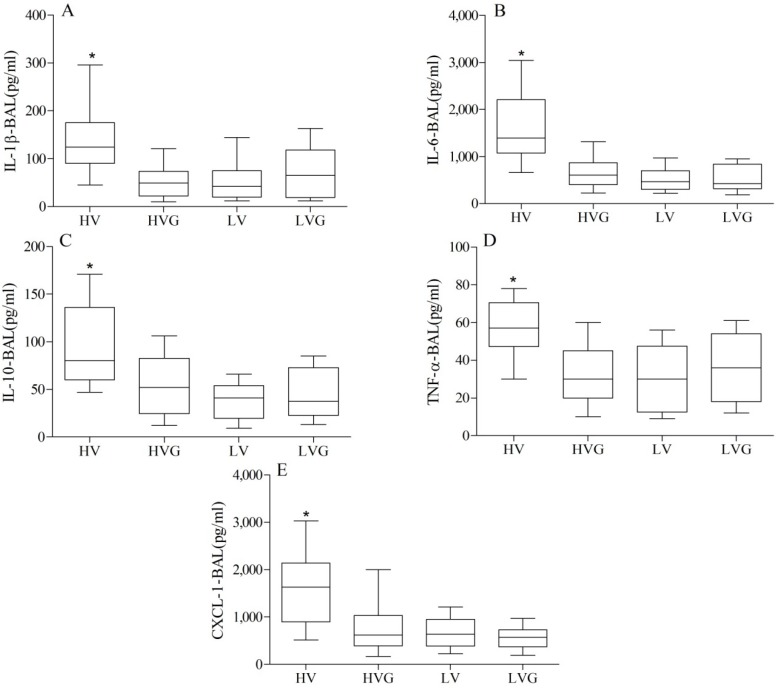
Cytokine levels in BAL. * *p* < 0.05 *vs.* other groups (*n* = 10/group). Rat groups: HV, acid aspiration + high TV; HVG, acid aspiration + high TV + glutamine; LV, acid aspiration + low TV; LVG, acid aspiration + low TV + glutamine.

The HV group had significantly higher plasma levels and BAL fluid levels of IL1-β, IL-6, IL-10, TNF-α, and CXCL1 compared to the other groups (Figure 4 and Figure 5). Again, GLN administration resulted in a significant reduction in this injury caused by a high TV.

### 3.4. Lung Histology

**Figure 6:**
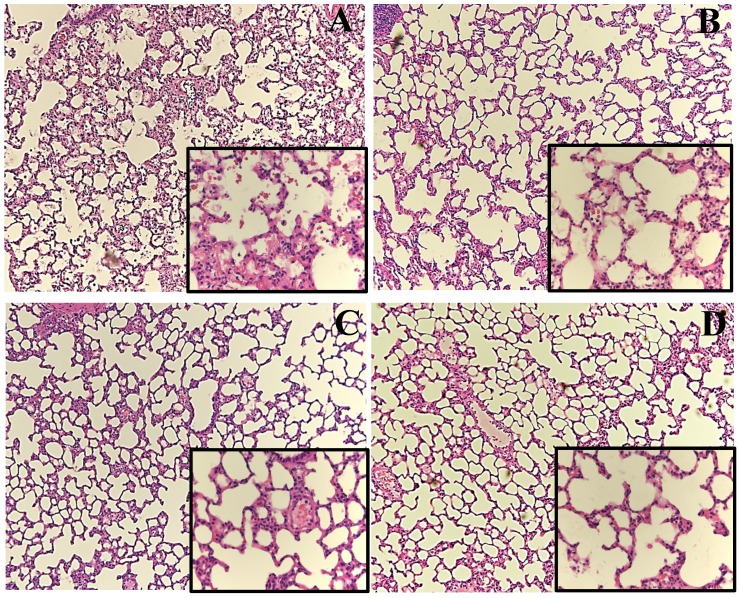
Representative lung histology slides (original magnification ×100/400). (**A**) HV, acid aspiration + high TV; (**B**) HVG, acid aspiration + high TV + glutamine; (**C**) LV, acid aspiration + low TV; and (**D**) LVG, acid aspiration + low TV + glutamine.

The lung histology results of the HV group showed aggravated neutrophil infiltration in the alveolar and interstitial spaces, hyaline membranes, proteinaceous debris that filled air spaces, and alveolar septal thickening as compared to the control group (Figure 6), as also indicated in Figure 3C. GLN administration had significantly attenuated this lung injury.

## 4. Discussion

In this study, the severity of aspiration-induced pulmonary inflammation and lung injury in a rat model was intensified followed by VILI. Generally speaking, mechanical ventilation is a lifesaving therapy as it improves hypoxemia and promotes alveolar opening in patients with acute respiratory failure; although, excessive alveolar distention as volutrauma and the cyclic opening and closing of lung units as atelectrauma may promote VILI [7]. Previous studies have shown that VILI alone may not cause extensive lung injury in the normal lung, but that VILI can enhance lung inflammation in pre-injured lungs [8,26]. This two-hit injury makes the lung more susceptible to mechanical ventilation injury, as there is increased pulmonary permeability, edema, neutrophil recruitment, and cytokine release into plasma [8,26]. It is known that a protective lung strategy (TV; 6 mL/kg) has survival benefits as compared to traditional TV (12 mL/kg) [10]. However, a low TV strategy may not be applicable for all patients with ARDS [11]. Thus, an effective pharmacologic intervention is required to treat these patients. We demonstrated the potential beneficial effects of GLN as reflected by diminished lung destruction, neutrophil recruitment, and “cytokine storms”.

These beneficial effects of GLN might be explained by the mechanism of reduced biotrauma from the cytokine responses and attenuation of neutrophil infiltration into the lung. Some cytokines may lead to pulmonary inflammation mediated by neutrophil recruitment, among which TNF-α and the CXC chemokine family are important [27]. TNF-α may prime neutrophils in response to specific activating factors that enhance respiratory bursts, release reactive oxygen metabolites, and induce phagocytosis [27]. Increased TNF-α levels were found to be associated with a higher incidence and severity of ARDS and mortality [28]. In addition, CXCL1, the equivalent of human IL-8, is a chemokine that recruits and activates all leukocytes and stimulates the release of IL-1, IL-6, and TNF-α from fibroblasts and macrophages, and during mechanical ventilation, lung epithelial cell stretch may contribute to elevated IL-8 levels [29]. The increased levels of IL-8 in ARDS patients with the subsequent aggregation of neutrophils play a pivotal role in the pathogenesis of ARDS [30].

Besides, IL-1β is an early, central pro-inflammatory cytokine that is released in response to stress. IL-6 is a pleiotropic cytokine with a wide range of biologic actions in acute and chronic inflammation, vascular disease, and cancer, and serum and BAL IL-6 levels are correlated with ARDS mortality [30]. The release of TNF-α, IL-1β, IL-6, and the CXC chemokine family is associated with neutrophil recruitment [31] and with the severity of ARDS and mortality [32]. Our findings were in agreement with these reports in that IL1-β, IL-6, TNF-α and CXCL1 levels in both plasma and the lung were potentiated by the injurious ventilator strategy in the HV group. The immune modulating properties of GLN could attenuate these effects, as shown in some previous studies [14,15,16,17,18].

In addition, GLN can modulate immune cell functions and reduces inflammation and the production of cytokines via the attenuation of NF-κB, kinases, proteins, iNOS expression, the interactions between neutrophils and the endothelium, and neutrophil infiltration into tissues [14]. Enhanced tissue GSH levels and heat shock protein levels caused by infusing GLN are partly responsible for preventing NF-κB activation, increased antioxidant capacity, and attenuating the release of TNF-α [23,24]. Enhanced heat shock protein expression is associated with cell survival under stressful conditions [17]. Furthermore, IL-10 can inhibit pro-inflammatory cytokines and the severity of an inflammatory insult and the concentrations of pro-inflammatory cytokines, such as IL-1β and TNF-α, are correlated with the magnitude of an endogenous IL-10 response [33]. A previous study showed that pre-treatment with GLN reduced IL-10 expression in human peripheral blood mononuclear cells stimulated by LPS, and that this was related to enhanced heat shock protein 70 expression [34]. Taken together, a better balance in cytokine profiles may have contributed to the beneficial effects in GLN-treated animals, as evidenced in the present study.

Neutrophil activation is largely responsible for lung injury, as it results in the production of cytokines and chemokines, oxidative bursts, and the release of proteolytic enzymes [10]. Intracellular oxidative stress causes vascular barrier disruption and pulmonary edema [35]. In addition, the major enzyme for GLN metabolism, glutaminase, is present in human neutrophils [36], which indicates that neutrophil function is modulated by GLN. Reduced levels of pro-inflammatory cytokines, such as IL-8, in the human gut are caused by inhibiting neutrophil activity as GLN utilization is increased [37]. In addition, supplementation with GLN significantly suppressed TNF-α production in LPS-stimulated neutrophils [38]. Therefore, our results suggest that GLN administration significantly suppressed neutrophil infiltration and activity, which is consistent with other models [39,40].

Other experimental work has also shown that GLN supplementation was beneficial for treating lung injuries induced by ischemia-reperfusion, endotoxin exposure, hyperoxia, smoke inhalation, and sepsis [17,41,42,43,44,45]. GLN supplementation might reduce the occurrence of infections, hospital lengths of stay, and mortality for severely ill patients [46] and reduced mortality and complications in burn patients with Gram-negative bacteremia [47]. However, conflicting results have also been reported [48]. Current guidelines by the European Societies of Parenteral and Enteral Nutrition (ESPEN) give GLN supplementation a grade A evidence level and states, ‘‘When PN is indicated in ICU patients the amino acid solution should contain 0.2–0.4 g/kg/day of l-glutamine’’ [49]. Our study results indicate that GLN may have been overlooked in ARDS.

There were some limitations to our study. Firstly, only one single i.v. dose of GLN (0.75 g/kg) was used [17,18,41,42,44], and it is possible that multiple doses, continuous infusion, or the enteral route as shown on similar acute lung injury study induced by oleic acid [50] could have yielded better histological results. Secondly, we did not measure GLN serum levels before and during the study period, although lower plasma GLN levels have been shown to be associated with increased mortality [51]. Thirdly, we did not check for heat shock proteins and GSH levels to establish the possible mechanisms of tissue protection, although our lung histology results provided evidence for this. Fourthly, the results obtained with an acid aspiration-induced experimental model in such a short timeline (4 h ventilator) may not be extrapolated to other experimental models of ARDS or to critically ill patients with various causes of ARDS other than aspiration pneumonia. Finally, the current study did not address issues of mechanism of action of GLN.

## 5. Conclusions

In conclusion, our study results showed that GLN administration improved the physiologic and biologic profiles of an experimental model used in VILI research based on the two-hit theory of acid aspiration-induced lung injury followed by injurious MV in rats. This was shown by the improvements in oxygenation and static compliance, and by the decreases in elastance, lung edema, neutrophil recruitment, extended lung destruction, and cytokine production. In clinical practice, MV should be carefully adjusted for patients with acid aspiration-induced lung injury. The hypothesis that GLN provides protective effects for lung injury will require further clinical studies.
